# RETRACTION: Effect of Different Surgical Techniques on Postoperative Wound Infection in Patients With Uterine Prolapse: A Meta‐Analysis

**DOI:** 10.1111/iwj.70386

**Published:** 2025-03-21

## Abstract

**RETRACTION:** WangJ. , ShangX. , HuangJ. , and WangJ. , “Effect of Different Surgical Techniques on Postoperative Wound Infection in Patients With Uterine Prolapse: A Meta‐Analysis,” International Wound Journal 21, no. 1 (2024): e14588, 10.1111/iwj.14588.38272813 PMC10794079

The above article, published online on 17 January 2024, in Wiley Online Library (http://onlinelibrary.wiley.com/), has been retracted by agreement between the journal Editor in Chief, Professor Keith Harding; and John Wiley & Sons Ltd. Following an investigation by the publisher, all parties have concluded that this article was accepted solely on the basis of a compromised peer review process. The editors have therefore decided to retract the article. The authors did not respond to our notice regarding the retraction.

**RETRACTION:** J. Wang , X. Shang , J. Huang , and J. Wang , “Effect of Different Surgical Techniques on Postoperative Wound Infection in Patients With Uterine Prolapse: A Meta‐Analysis,” International Wound Journal 21, no. 1 (2024): e14588, 10.1111/iwj.14588.38272813 PMC10794079

The above article, published online on 17 January 2024, in Wiley Online Library (http://onlinelibrary.wiley.com/), has been retracted by agreement between the journal Editor in Chief, Professor Keith Harding; and John Wiley & Sons Ltd. Following an investigation by the publisher, all parties have concluded that this article was accepted solely on the basis of a compromised peer review process. The editors have therefore decided to retract the article. The authors did not respond to our notice regarding the retraction.

